# High expression of NDRG3 in osteoarthritis patients

**DOI:** 10.1186/s42836-020-00064-2

**Published:** 2021-03-01

**Authors:** Long Chen, Yuanzheng Wang, Senlei Li, Wei Zhou, Li Sun

**Affiliations:** 1 Department of Orthopedics, Guizhou Provincial People’s Hospital, 550000 Guiyang, Guizhou China; 2 Department of Orthopedics, People’s Hospital of Yunyan District, 550000 Guiyang, Guizhou China

**Keywords:** NDRG3, HIF-1α, HIF-2α, Osteoarthritis, Pathogenesis

## Abstract

**Background:**

Osteoarthritis (OA), as a common disease, seriously affects the quality of life of the victims, but its pathogenesis remains unclear. It has been confirmed that hypoxia-induced factor (HIF)-mediated hypoxia response plays an important role in the development and progression of OA. As a member of the N-myc downstream regulatory gene families, NDRG3 has been reported to independently regulate the hypoxic response of tumour cells, but the relationship between NDRG3 and OA development has not been reported so far.

**Methods:**

In this study, seven OA patients were admitted to Guizhou Provincial People’s Hospital from January 2017 to December 2018. The OA group included 5 patients clinically diagnosed with hip/knee OA, which required arthroplasty. The normal group included 2 patients with no previous history of OA and rheumatoid arthritis, which required amputation due to trauma or tumour. The articular cartilage samples were collected to detect the expression of HIF-1α, HIF-2α and NDRG3 using immunohistochemical (IHC), haematoxylin and eosin (HE) and toluidine blue (TB) staining.

**Results:**

HE and TB staining indicated that the cartilage surface of the normal group was smooth and intact, with a columnar arrangement of hyaline chondrocytes, while the cartilage surface of the OA group was discontinuous, with cartilage missing and fibrous soft tissue growing into the defect site. HIF-1α staining was positive in both groups. Moreover, HIF-2α and NDRG3 staining was weakly positive in the normal group, but were uniformly and strongly positive in the OA group. The positively stained areas and integral optical density for NDRG3 were significantly greater in OA group than in the normal group (*p* < 0.05).

**Conclusions:**

NDRG3 might be closely related to the development and progression of OA. However, the relationship between NDRG3 and OA, which is independent of the HIF pathway, warrants further research.

## Introduction

Osteoarthritis (OA), orthopaedically the most common chronic degenerative disease, mostly occurs in the hip and knee joints that are constantly subjected to heavy loads and stretching. The main symptoms of OA are joint pain and stiffness, and severe cases may have joint deformities. It poses a heavy burden on patients’ family, healthcare system and the society at large and is a major medical problem that needs to be addressed [1]. Recently, epidemiological data showed that the prevalence of knee OA in China is as high as 8.1%, involving over 100 million patients, and the incidence increases with age, which presents a huge challenge for future prevention and control of the condition [2]. Current research has found that articular cartilage degeneration caused by metabolic disorders of chondrocytes is an important initiating factor and the major pathological change of OA [3–5]. Chondrocyte transcriptome changes are not obvious at early stages of OA. Nonetheless, dramatic changes occur during the late stages, resulting in upregulation of a variety of genes related to extracellular matrix decomposition and downregulation of genes related to oxidative damage defence [6, 7]. All these render early diagnosis of OA very difficult. OA is often diagnosed in the middle and at late stages, and treatment at this time is challenging. Therefore, the mechanism of chondrocyte degeneration has been a subject of active research in OA studies, and understanding of the underlying mechanism can provide therapeutic targets for the prevention and treatment of OA.

The hypoxic response mechanism plays an important part in various tissues, organs and lesions of the human body [8]. Normal articular cartilage is a non-vascular tissue under physiological conditions, and its nutrition and oxygen supply mainly depend on the spread of synovial fluid and subchondral bone. Articular cartilage tissue is a hypoxic environment, and studies have shown that its oxygen content is about 1–5%. Its oxygen content gradually decreases from the surface of the cartilage down to the deeper layers [9]. Hypoxia-inducible factor (HIF) is a key regulatory factor produced by cells in response to a hypoxic environment. At present, most research efforts are directed to the effects of HIF-1α and HIF-2α on cartilage degeneration [10]. Previous researches have found that, under physiological conditions, chondrocytes can elicit the expression of a variety of genes related to cartilage matrix synthesis and transport (such as COL2A1, AGC1 and SOX9) by activating the HIF-1α pathway to maintain the stability of articular cartilage matrix and energy metabolism [11]. Presumably, HIF-1α is a key regulator of articular cartilage adaptation to a hypoxic environment. During the development of OA, after the activation of the HIF-2α pathway, the expression of a variety of genes that regulate cartilage matrix decomposition and inflammatory responses are up-regulated, including NF-κB, MMP-3, MMP-13, IL-6 and RUNX2. At the same time, disordered synthesis and decomposition of chondrocytes and imbalances in energy metabolism also ensue. These factors jointly lead to the degeneration of articular cartilage [12]. Therefore, an in-depth study of the expression of related molecules under a hypoxic environment in cartilage not only helps us better understand the development and progression of OA, but also provides guidance to clinical diagnosis and treatment, and drug development. The OA studies on hypoxia response-regulating mechanism of articular chondrocytes focused on the HIF family alone. Understanding whether chondrocytes have other hypoxic response-regulating mechanisms in the development of OA is of considerable significance to the further characterization of the regulatory network of OA in a hypoxic environment.

NDRG3 is a member of the N-myc downstream-regulated gene (NDRG) family and is located on the human chromosome 20q11.21-11.23. The NDRG-cDNA is 2588 bp in length and contains an open reading frame. Its relative molecular mass is 40 kD [13]. NDRG3 is involved in multiple biological regulatory processes, such as cell proliferation and differentiation in organisms [14]. It also plays a key regulatory role in the tumour cell proliferation [13], vascular endothelial renewal [15] and DNA damage repair in meiosis [16]. More interestingly, NDRG3 has been shown to be closely related to the regulation of tumour cells in a hypoxic environment [17]. Lee et al. demonstrated that NDRG3 could regulate the hypoxic response of tumour cells independent of the HIF pathway. Under hypoxic conditions, the lactate produced by glycolysis binds to NDRG3, in which the ubiquitination degradation of NDRG3 by PHD2/VHL is prevented and NDRG3 expression is up-regulated. Up-regulated expression of NDRG3 then activates the Raf-ERK pathway and thereby promotes cell proliferation and angiogenesis, eventually resulting in tumour growth and progression [18]. This suggests that signal pathways involved in the hypoxic response of cells were much more complex than previously known. In the course of OA, not only are the known classical regulators or pathways involved, but more intertwined or independent regulatory pathways are also implicated.

On the basis of the NDRG3-mediated hypoxic response, we surgically collected specimens from normal and OA patients, and conducted a variety of histopathological experiments to explore the relationship between NDRG3 and the development and progression of OA.

## Materials and methods

### Study design

A restrospective evaluation was performed on seven OA patients from January 2017 to December 2018. The general information of the patients is listed in Table 1. The normal group included 2 patients who needed amputation due to trauma and tumour (articular cartilage was taken from the elbow, wrist and ankle respectively). The OA group included 2 patients with hip OA and 3 patients with knee OA. In hip/knee arthroplasty of the OA patients, the articular cartilages were taken in the OA group. In other amputations, articular cartilage in the amputated limbs without lesions and from accumulated joints were taken in the normal group. This study was approved by Guizhou Provincial People’s Hospital, and informed consent was obtained from all participants.

**Table 1:** General information of the patients

Patients	Age (year)	Gender	Diagnosis	Surgical operation	Sample (cartilage)
1	34	Male	Open fracture of right humerus	Right upper arm amputation	Elbow and wrist
2	52	Male	Malignant fibrous histiocytoma of the left femoral condyle	Left thigh amputation	Ankle
3	71	Female	Bilateral knee osteoarthritis	Right knee replacement	Knee
4	59	Female	Bilateral knee osteoarthritis	Left knee replacement	Knee
5	70	Female	Left knee osteoarthritis	Left knee replacement	Knee
6	75	Male	Right hip osteoarthritis	Right hip replacement	Hip
7	70	Female	Left hip osteoarthritis	Left hip replacement	Hip

### Histological staining and related analysis

Articular cartilage specimens from OA and normal groups were fixed with paraformaldehyde, decalcified, embedded and sectioned before immunohistochemical (IHC) staining with HE, toluidine blue (TB), HIF-1α, HIF-2α and NDRG3.

#### Haematoxylin and eosin (HE) staining

First, we dewaxed the prepared slides and stained them with haematoxylin for 10–20 min, then differentiated them with HCl and alcohol for 5–10 s, and rinsed with tap water for 1–3 min before and after differentiation. Afterwards, they were put into warm water at 50 °C, or weak alkaline solution, rinsed with tap water for 1–3 min until blue color appeared, then soaked in 85% alcohol for 3–5 min, stained with eosin for 3–5 min, dehydrated with gradient alcohol after washing for 3–5 s. Finally, we permeated and sealed samples using xylene and neutral gum separately.

#### TB staining

Firstly, we performed the anti-shedding treatment on the slides. The slides were soaked with APES, then removed of APES and incubated at 60 °C for 60 min to make the sections adhere tightly. Then, the sections were routinely dewaxed and placed in a 1% TB aqueous solution pre-heated to 50 °C, dyed in a 56 °C incubator for 20 min, washed with distilled water, soaked in 70% alcohol for 1 min, and differentiated in 95% alcohol. Finally, under a microscope, when the Nissl bodies were clearly displayed, dehydration was quickly performed using anhydrous alcohol. Then the slides were permeated and sealed with xylene and neutral gum separately.

#### HIF-1α (Proteintech, USA), HIF-2α (SAB, USA) and NDRG3 (SAB, USA) IHC staining

Firstly, the dewaxed slides were placed in a staining tank, soaked in 3% methanol hydrogen peroxide for 10 minutes at room temperature, washed 3 times with PBS for 5 min each time, and then immersed into 0.01 M citrate buffer solution, and heated to boiling point in a microwave after a 5 min interval. After cooling, the slides were washed twice with PBS for 5 min each, then blocked with goat serum for 20 min at room temperature, incubated with the primary antibody at 4 °C overnight and biotinylated with secondary antibody at 37 °C for 30 min. Next, streptavidin reagent (wet box, labelled with alkaline phosphatase) was added and the resultant sample was incubated at 37 °C for 30 min, washed thoroughly with water, and then developed for DAB colour for about 2 min using a DAB kit (Thermo Fisher Scientific, USA). Finally, the slides were washed with distilled water, and then slightly counterstained with haematoxylin, dehydrated, permeated and sealed.

#### Positively-stained area and integrated optical density

The pathological slides were scanned and read on a fully automatic radiographer (NatureGene Corp, USA). Four pieces were randomly selected from each specimen under a 40× field of view. Image-Pro Plus (Vision 6.0) software (Media Cybernetics, USA) was used to calculate the positively-stained area and integrated optical density (IOD).

### Statistical Analysis

We employed GraphPad Prism 6 software package (GraphPad Software Inc., USA) for statistical analysis. Quantitative data were expressed as mean ± SD, and differences between two groups were analyzed by using Student’s *t* test. A *p* < 0.05 indicated a statistically significant difference.

## Results

### HE staining

**Fig. 1:**
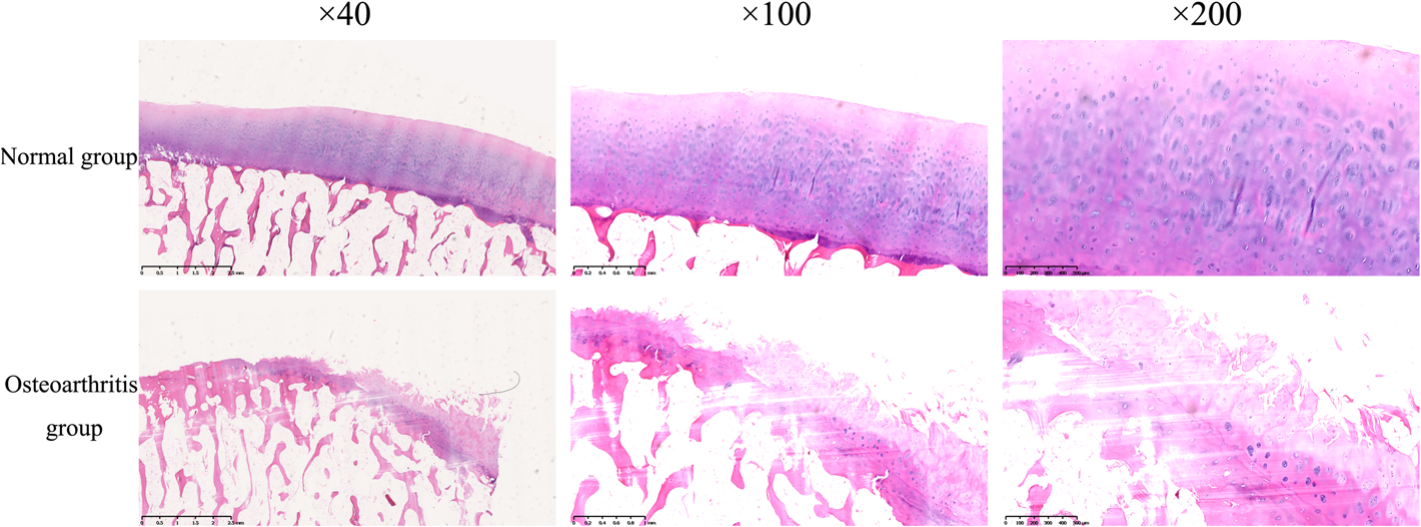
The HE staining of the cartilage in the normal group and the osteoarthritis group

The cartilage surface of the normal group was smooth and intact with a columnar arrangement of hyaline chondrocytes. The matrix showed a uniform basophilic staining, and the chondrocytes a strong basophilic staining. The cartilage surface of the OA group was discontinuous, with the cartilage missing and fibrous soft tissue growing into the defect site (Fig. 1).

### TB staining

**Fig. 2:**
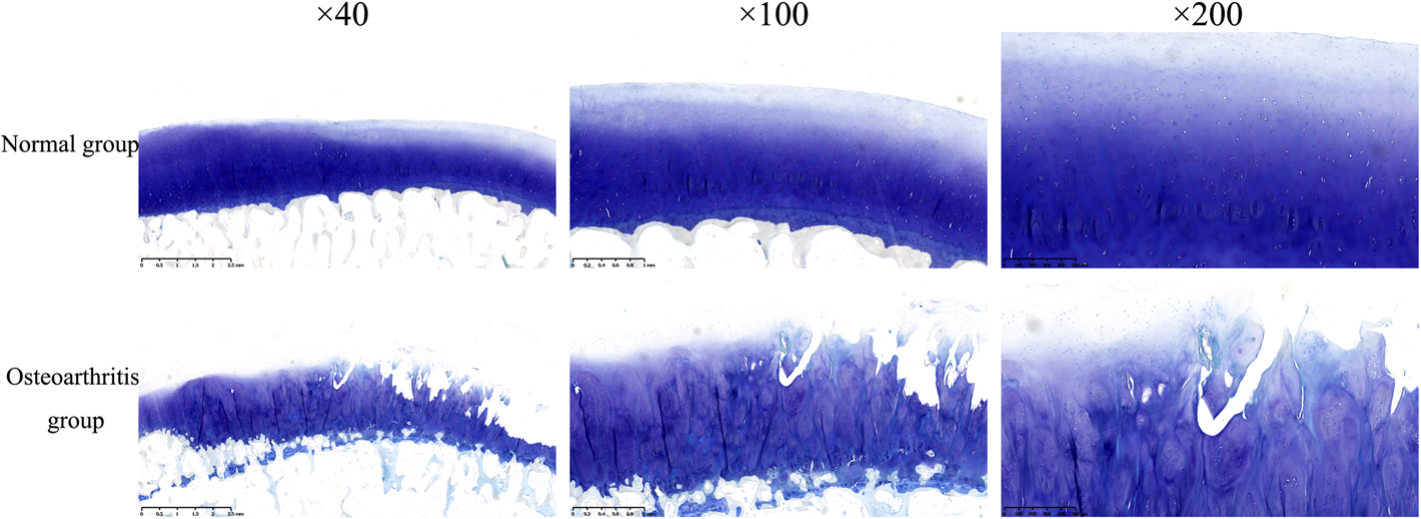
The Toluidine blue staining of the cartilage in the normal group and the osteoarthritis group

The cartilage surface in the normal group was smooth and intact with a columnar arrangement of hyaline chondrocytes. The nuclei were clearly stained blue, while the cytoplasm was pale blue-purple. The cartilage surface of the OA group was discontinuous and had a vacuole-like structure, with the cartilage missing and fibrous soft tissue growing into the defect site (Fig. 2).

### HIF-1α staining

**Fig. 3:**
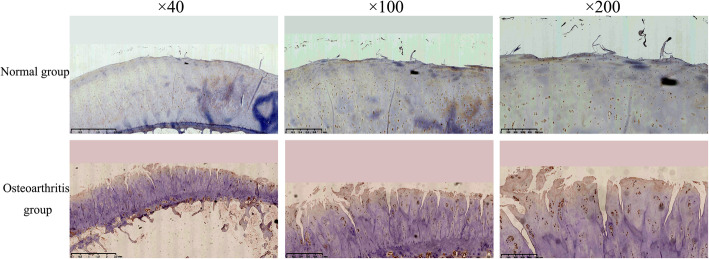
The HIF-1α staining of the cartilage in the normal group and osteoarthritis group

The chondrocytes in the normal group stained positive, and the immunopositive staining was extensive and stronger in the OA group than that in the normal group (Fig. 3).

### HIF-2α staining

**Fig. 4:**
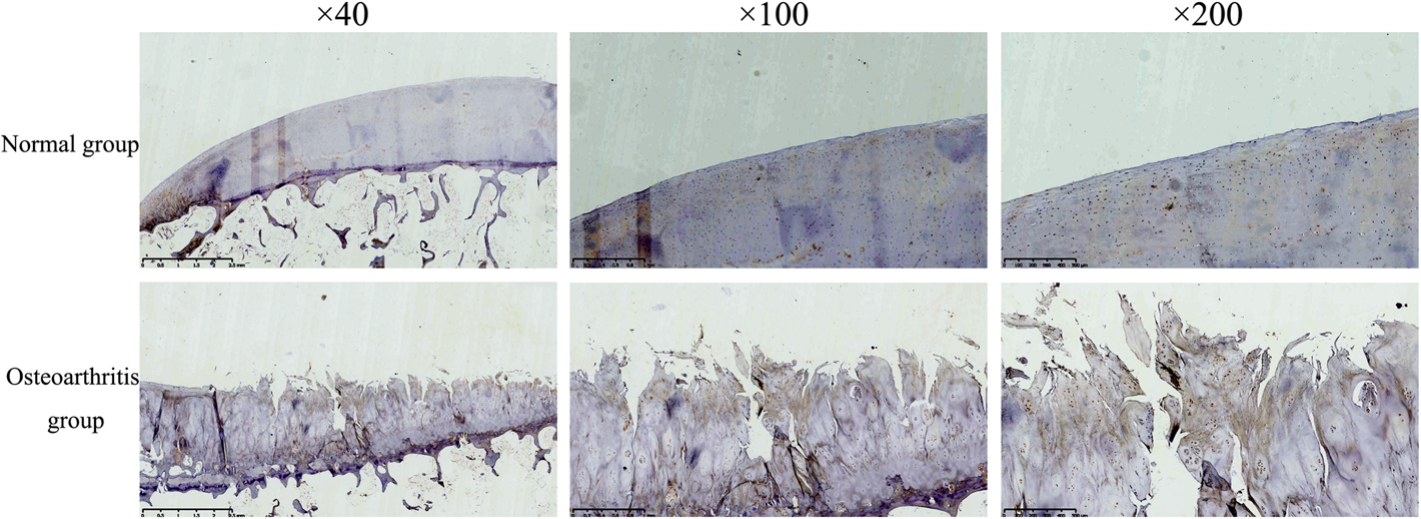
The HIF-2α staining of the cartilage in the normal group and osteoarthritis group

The chondrocytes in the normal group were weakly stained, and the OA group showed uniform and obvious positive immunostaining (Fig. 4).

### NDRG3 and related analysis

**Fig. 5:**
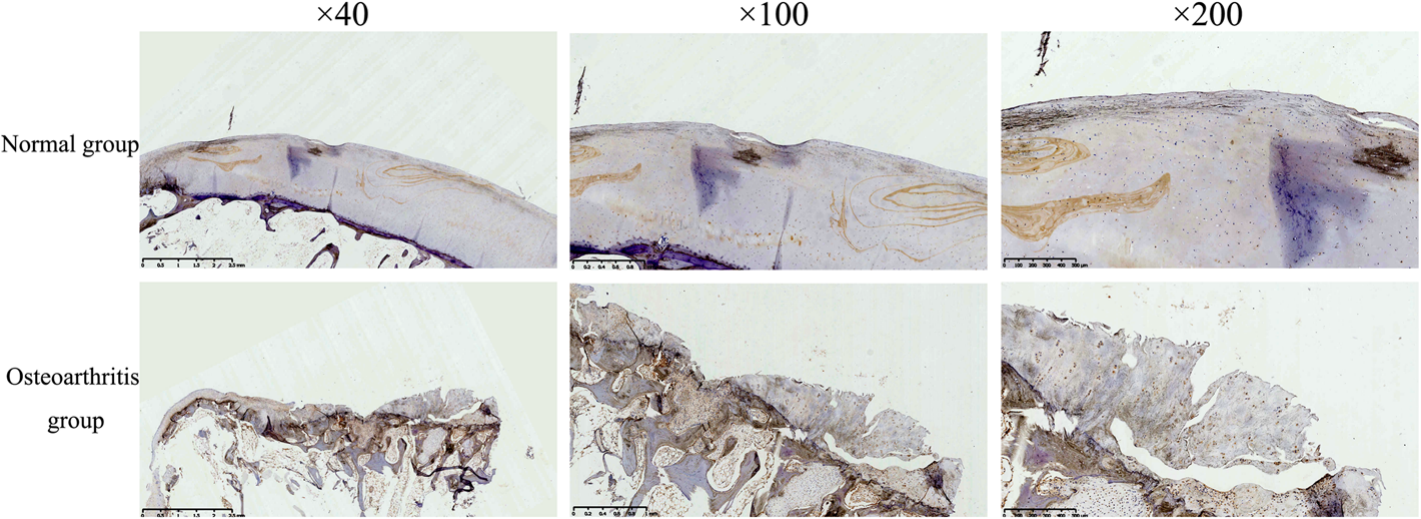
The NDRG3 staining of the cartilage in the normal group and osteoarthritis group

**Fig. 6:**
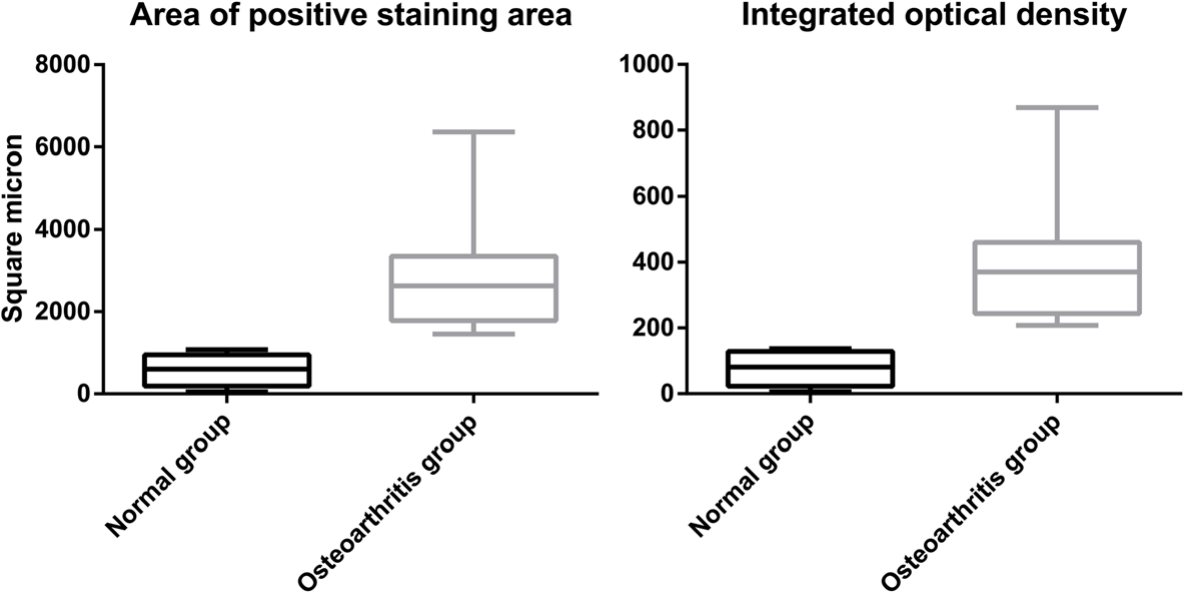
Statistical results of the area of positively-stained area and the integrated optical density for NDRG3 in the normal group and osteoarthritis group

The cartilage cells in the normal group were stained weakly, and the cells in the OA group was obviously immuno-positive and uniformly stained (Fig. 5). The statistical analysis showed that the positive staining area and integrated optical density were significantly greater in the OA group than in the normal group, and the differences were statistically significant (*p* < 0.05) (Fig. 6).

## Discussion

Articular cartilage, consisting of rich cartilage matrices and a low proportion of chondrocytes, is translucent and plays a role in the lubrication and cushioning of joints. The cartilage matrix, mainly composed of a network of type II collagen fibers, is filled with amorphous chondroitin sulphate in the voids where the chondrocytes are located.

Chondrocytes are unique cellular components that make up articular cartilage. Under normal circumstances, chondrocytes can maintain cartilage homeostasis by balancing the synthesis and decomposition of proteins, especially extracellular matrix type II collagen and proteoglycan [19]. However, in OA, the normal metabolic balance is upset, and the synthesis of type II collagen and proteoglycan is impaired, and the decomposition is accelerated [20]. The synthesis of various types of collagen (type I and type X) is up-regulated, thereby changing the ambient environment and physiological state of the chondrocytes, inducing apoptosis and leading to development and progression of OA [5]. Because articular cartilage lacks blood supply and the joint cavity is in a relatively closed state, the articular cartilage tissue *per se* is a hypoxic environment, and its oxygen content is about 1–5%, which gradually decreases down from the cartilage surface to the deeper areas [6]. Therefore, under physiological conditions, chondrocytes produce a series of hypoxia-related molecules to adapt to this environment, and these molecules participate in the regulation of the dynamic balance of chondrocyte proteins, autophagy, apoptosis, thereby contributing to the development and progression of OA.

HIF protein, comprising of α and β subunits, belongs to the transcription factor family and is a key regulator of cell response to hypoxia. The current research on hypoxia response mainly focused on HIF-1 and HIF-2. Under normal oxygen conditions, proline and asparagine of HIF-α are hydroxylated by prolyl hydroxylases (PHD), and then activated by Von Hippel-Lindau protein (pVHL)-mediated ubiquitination, which eventually leads to degradation of HIF-α. While under hypoxic conditions, HIF-α, which is not hydroxylated, and β subunits are heterodimerised and enter the nucleus to activate targeted genes and produce corresponding biological effects. The latest studies showed that HIF-1α played an important protective role in the maintenance of the homeostasis of the articular cartilage matrix, while HIF-2α had a negative decomposing effect [11]. HIF-1α has multiple responses when chondrocytes respond to hypoxia. It restricts the expression of matrix metallopeptidase-13 (MMP13) and inhibits the catabolism of chondrocytes by regulating the wnt signalling pathway [21, 22]. It can also regulate collagen (increase COL2A1 and inhibit COL1A1, COL1A2 and COL3A1) and proteoglycan expression to increase the synthesis of the cartilage extracellular matrix [23]. Moreover, HIF-1α also protects articular cartilage by regulating the apoptosis of chondrocytes [24–28]. HIF-2α, as an important regulator of cartilage tissue catabolism in an hypoxic environment, is mainly found in highly-differentiated chondrocytes, and can induce chondrocytes to synthesize a wide array of key factors, such as MMP1, 3, 9, 12 and 13, protein aggregation carbohydrase-1, -2, nitric oxide synthase-2 and cyclooxygenase-2 [29, 30]. In addition, HIF-2α is thought to be involved in early angiogenesis and to play an important part in osteogenesis in cartilage [31]. HIF-2α also inhibits autophagy in the chondrocyte maturation, although HIF-1α mainly plays a promoting role [9, 32]. Studies have found that the content of HIF-2α in OA cartilage tissues in mice and humans was significantly increased, and treatment regimens targeting HIF-2α are now under increasingly active studies. Zhang et al. found that chondroitin-1 could maintain cartilage homeostasis by inhibiting the degradation of HIF-2α [12]. Pi et al. used nanoparticles coated with HIF-2α siRNA to treat murine OA and achieved good results [29]. HIF-2α is also implicated in the pathological process of OA. In this study, HIF-1α was found to be expressed in both groups, while HIF-2α was significantly increased in the OA group, suggesting that HIF-1α maintains the metabolic balance of chondrocytes, and HIF-2α is involved in the development and progression of OA. Our research also confirmed the findings of previous cell and animal experiments.

**Fig. 7:**
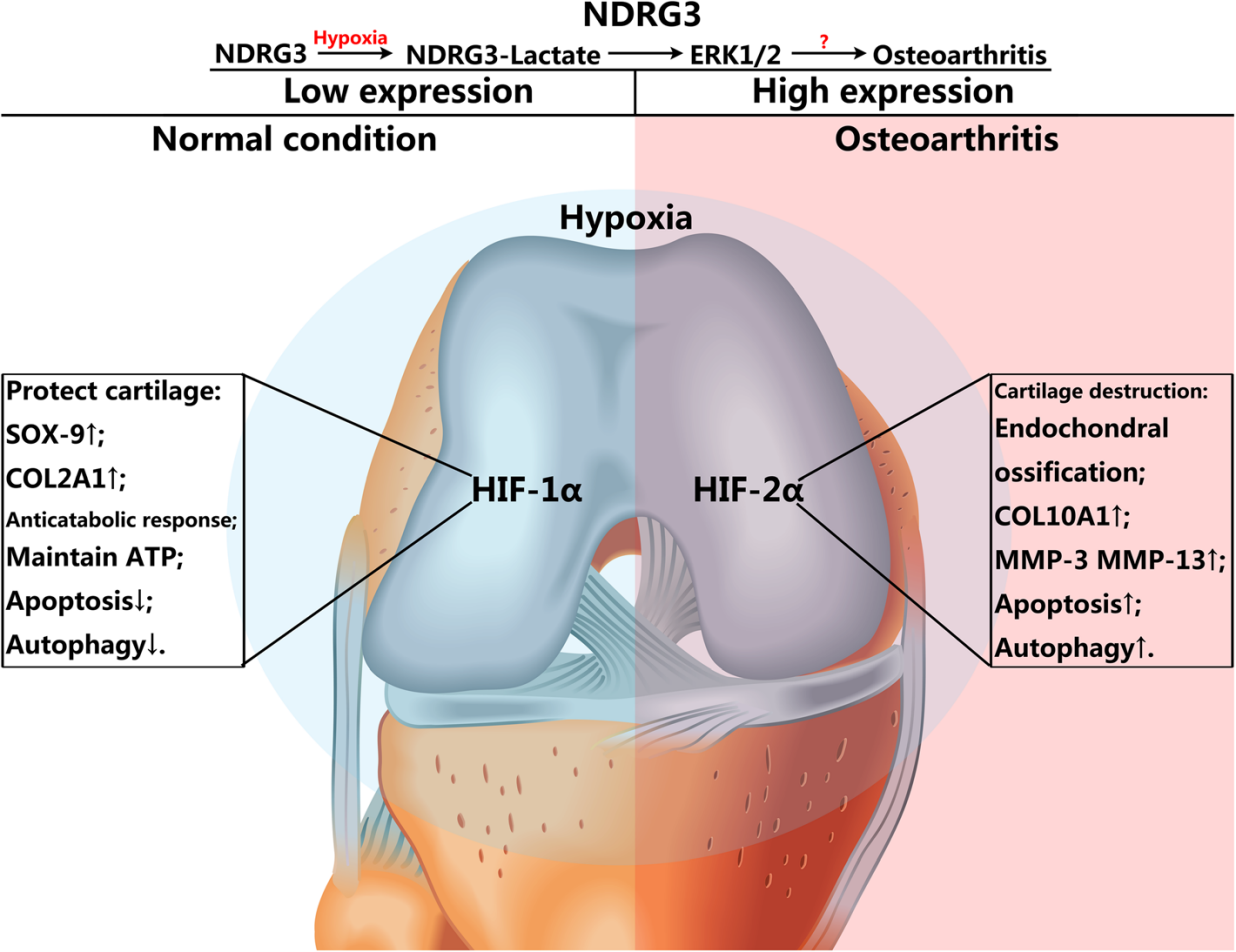
NDRG3 may be closely related to the development and progression of osteoarthritis

NDRG3, as a member of the N-myc downstream regulatory gene family, exists in a variety of human organs, such as ovary, prostate, testes, brain, spinal cord, thymus, heart and kidney. Lee et al. found that under normal circumstances, NDRG3 was degraded through the PHD2/VHL pathway; but under hypoxic conditions, NDRG3 could bind to lactic acid and c-Raf protein, respectively, and promote angiogenesis and cell growth through the Raf-ERK pathway [18]. Yao et al. found that, in a model of cerebral ischemia, *in vitro* and *in vivo*, let-7f could increase local tissue vascular regeneration and cell survival by regulating the expression of NDRG3, and the let-7f/NDRG3 pathway could be used as a target for the treatment of ischemic stroke [33]. The joint cavity has a special hypoxic environment and no studies were conducted on NDRG3-mediated hypoxic response in the pathogenesis of OA. Our study found that NDRG3 expression in the OA group was significantly higher than that in the normal group, which suggested that an NDRG3-mediated hypoxia response may be intimately related to the development and progression of OA. Nonetheless, its specific molecular mechanism remains to be confirmed. The results of this study indicated that cell signalling pathways are complicated under hypoxic conditions. Similarly, not only classic regulatory factors or pathways but also other factors may play an important role in the development and progression of OA (Fig. 7).

This study has several limitations which should be noted. Firstly, due to the small sample size of each group, accuracy may have been reduced. Secondly, the study included no laboratory tests, especially at the cellular and molecular levels, so the relation between HIF-1α, HIF-2α and NDRG3 remains uncertain. Finally, we included patients with osteoarthritis (hip or knee) and the cartilage was harvested from hip and knee, which might generate heterogeneity.

## Conclusions

In summary, HIF-1α can maintain the balance of chondrocyte breakdown and anabolic metabolism by regulating the expression of various proteins. At the same time, HIF-2α is involved in the pathological process of OA and is a key regulator of articular cartilage degeneration. NDRG3, which mediates the hypoxic response independent of the HIF pathway, might be closely related to the development and progression of OA, and its relationship with OA warrants to be further explored and confirmed in the future.

## Data Availability

Not applicable.

## Abbreviations

OA: Osteoarthritis
HIF: Hypoxia-induced factor
IHC: Immunohistochemical
HE: Haematoxylin and eosin
TB: Toluidine blue
NDRG: N-myc downstream-regulated gene
PHD: Prolyl hydroxylases
pVHL: Von Hippel–Lindau protein
MMP13: Matrix metallopeptidase-13
